# Real-time tunable lasing from plasmonic nanocavity arrays

**DOI:** 10.1038/ncomms7939

**Published:** 2015-04-20

**Authors:** Ankun Yang, Thang B. Hoang, Montacer Dridi, Claire Deeb, Maiken H. Mikkelsen, George C. Schatz, Teri W. Odom

**Affiliations:** 1 Department of Materials Science and Engineering, Northwestern University, Evanston, Illinois 60208, USA; 2 Department of Physics, Duke University, Durham, North Carolina 27708, USA; 3 Department of Chemistry, Northwestern University, Evanston, Illinois 60208, USA; 4 Department of Electrical and Computer Engineering, Duke University, Durham, North Carolina 27708, USA

## Abstract

Plasmon lasers can support ultrasmall mode confinement and ultrafast dynamics with device feature sizes below the diffraction limit. However, most plasmon-based nanolasers rely on solid gain materials (inorganic semiconducting nanowire or organic dye in a solid matrix) that preclude the possibility of dynamic tuning. Here we report an approach to achieve real-time, tunable lattice plasmon lasing based on arrays of gold nanoparticles and liquid gain materials. Optically pumped arrays of gold nanoparticles surrounded by liquid dye molecules exhibit lasing emission that can be tuned as a function of the dielectric environment. Wavelength-dependent time-resolved experiments show distinct lifetime characteristics below and above the lasing threshold. By integrating gold nanoparticle arrays within microfluidic channels and flowing in liquid gain materials with different refractive indices, we achieve dynamic tuning of the plasmon lasing wavelength. Tunable lattice plasmon lasers offer prospects to enhance and detect weak physical and chemical processes on the nanoscale in real time.

## Abstract (web summary)

Plasmonic lasers offer ultrasmall mode confinement via nanoscale structures, but their reliance on solid-state gain media makes tunability difficult. Yang *et al*, present a laser based on gold nanoparticle arrays in a microfluidic channel, whose liquid gain media enable dynamic tuning of the lasing wavelength.

## Graphical abstract

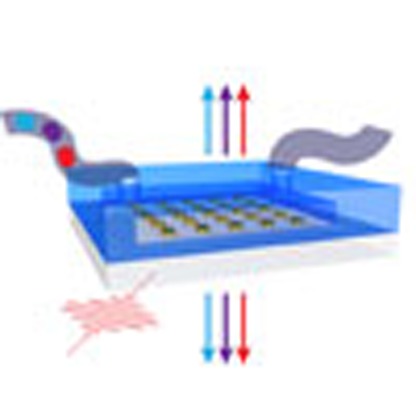

The integration of coherent light sources into optical circuits is enabling lab-on-a-chip systems for enhanced light–matter interactions and spectroscopic applications^1^,^2^. Wavelength tunability of the emission is a critical driver for practical applications. Distributed feedback (DFB) lasers based on waveguided dielectric structures can show low-threshold and high-output light emission, and lasing wavelengths can be tuned by changing the periodicity of the structures and the thickness of the gain layer^3^,^4^. However, DFB lasers have low modulation speeds^5^ and require index modulation to achieve mode confinement^3^,^6^. In contrast, plasmon nanolasers support ultrafast dynamics and ultrasmall mode volumes with device feature sizes below that of the diffraction limit^7^,^8^,^9^,^10^,^11^. Plasmon lasing via localized surface plasmons (LSPs) in nanoparticles (NPs) suspended in solution^9^ and surface plasmon polaritons on flat films^10^,^11^,^12^ often lack far-field beam directionality because of the large momentum mismatch between the nanolocalized lasing fields and free-space light. The arrangement of plasmonic resonators in a two-dimensional array has been proposed as a means to achieve directional lasing normal to the array plane via the in-phase oscillations of trapped-mode currents^13^. Recently, we discovered that room-temperature lasing emission with high directionality could be achieved based on band-edge lattice plasmons of periodic arrays of metal NPs^14^. Although there have been a few reports on tuning plasmon nanolasers by changing the doping levels of the semiconductor gain media^12^ or the emission properties of dye molecules^15^, these approaches are based on solid gain materials such as inorganic nanowires or organic dye in a solid matrix that precludes possibilities of dynamic tuning^9^,^12^,^14^,^16^.

Here we report how lasing action based on band-edge lattice plasmons can be tuned in real time by modulating the refractive index environment around the plasmonic NP arrays. We find that liquid gain, where IR-140 dye molecules are dissolved in organic solvents, shows superior lasing characteristics compared with the same dye in a solid matrix^14^. The plasmon–exciton energy transfer exhibits distinct lifetime characteristics below and above the lasing threshold, in good agreement with semi-quantum calculations using a four-level gain system and a time-domain approach. We design plasmonic cavities with a fixed NP size and lattice spacing that produce narrow lattice plasmon resonances, which can be tuned over a 55-nm wavelength range simply by changing the dielectric environment. Establishing tunability of the lattice plasmon is critical for lasing since the lasing emission is at the band-edge wavelength. Thus, liquid gain is created by dissolving the dye in different organic solvents to index-match the superstrate with the substrate, which enables the lasing signal to be tuned over the entire dye bandwidth. Finally, we exploit the liquid phase of the gain to realize a dynamically tunable plasmon laser by integrating the Au NP arrays into a microfluidic channel. Real-time switching and shifting of the nanolasing wavelength are achieved by flowing different plugs of liquid gain material through the channel.

## Results

### Designing a plasmon nanolaser with liquid gain

**Figure 1:**
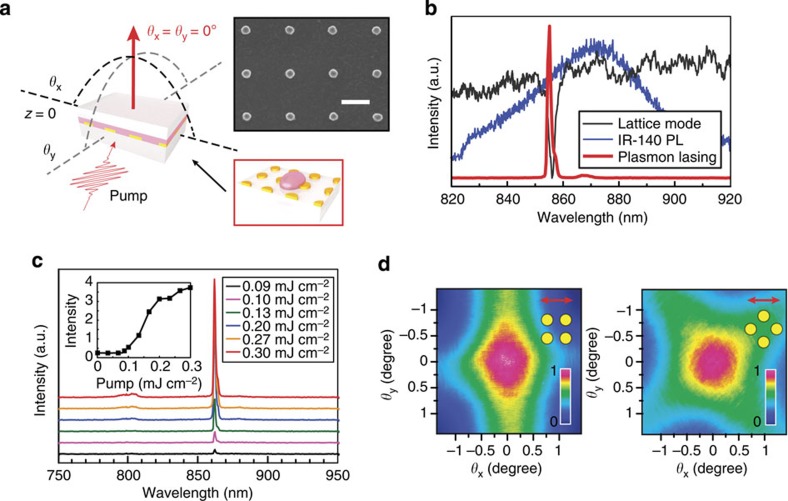
Lasing emission of Au NP arrays on fused silica with IR-140 in DMSO as superstrate. (**a**) Scheme of a lasing device and scanning electron microscopic image of Au NP arrays. Lasing devices consist of Au NP arrays on transparent substrates sandwiched between IR-140 dissolved in an organic solvent and a glass coverslip. Scale bar, 400 nm. (**b**) Lasing observed at the band edge where the IR-140 photoluminescence (PL) in DMSO overlapped with the lattice plasmon resonance. (**c**) Input–output of the lasing emission. (inset) Output intensity as a function of the pump energy. (Note: the different lasing wavelengths for **b** and **c** were from measurements on two different samples.) (**d**) (left) Measured far-field beam profiles when the Au NP arrays were pumped with polarization along the high-symmetry direction and (right) 45° with respect to the high-symmetry direction. The insets indicate polarization of the pump beam with respect to the Au NP arrays.

Figure 1a depicts a plasmonic nanocavity laser based on a two-dimensional Au NP array and liquid gain media. Liquid superstrates enable dye molecules to be solubilized and distributed more uniformly compared with cured polymer superstrates^14^,^17^. We used different solvents to dissolve IR-140 to create liquid gain. Dimethyl sulfoxide (DMSO) with refractive index *n*=1.48 was used to match the index of fused silica (*n*=1.46), a common substrate for Au NPs. We constructed lasing devices by sandwiching a droplet of IR-140-DMSO liquid (0.25 mM) between Au NP arrays on fused silica and a glass coverslip. The structures were optically pumped using an 800-nm femtosecond (fs)-pulsed laser, and the emission spectra were collected normal to the sample surface (Methods). At low pump intensities (<0.1 mJ cm^−2^), the emission showed a profile similar to that of the photoluminescence (PL) on the IR-140-DMSO-only control (full-width at half-maximum (FWHM) ∼50 nm) (Fig. 1b). Above a critical pump intensity (∼0.1 mJ cm^−2^), a sharp and intense emission peak (*λ*_lasing_=855 nm, FWHM=1.5 nm) emerged close to the position of the lattice plasmon resonance (*λ*=856 nm, FWHM=4 nm). The lasing emission was orders of magnitude stronger than the background spontaneous emission (SE). The pump energy versus output intensity curve exhibited a characteristic threshold behaviour with a marked change in slope around 0.1 mJ cm^−2^ (Fig. 1c) that was independent of IR-140 concentration (Supplementary Fig. 1a). In contrast, linewidth broadening was observed when the IR-140 concentration increased (Supplementary Fig. 1b). We hypothesize that this broadening has contributions from incoherent processes (for example, dephasing effects) associated with increased dye densities around the Au NPs. When pump powers exceeded 0.3 mJ cm^−2^, the lasing intensity dropped. We attribute this decrease to a combination of bleaching of the dye molecules, reduced dye integrity from local heating of the metal NPs and competing energy loss channels from amplified spontaneous emission (ASE) induced by propagating lattice plasmons^14^.

**Figure 2:**
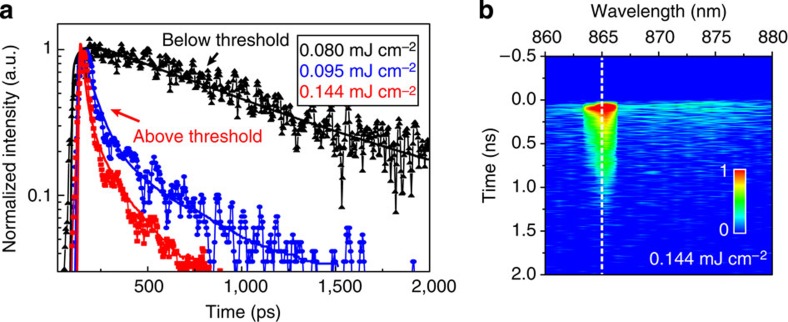
Time-correlated single-photon counting shows lifetime reduction above the lasing threshold. (**a**) Decay times measured at pump intensities below and above threshold show a reduction in lifetime around threshold (0.1 mJ cm^−2^) for the 865-nm lasing mode. The solid lines are fits to the data deconvolved with the instrument response function (IRF). (**b**) Lifetime map as a function of emission wavelength collected normal to the sample surface above threshold at 0.144 mJ cm^−2^. The scale bar indicates emitted photon intensity, increasing from blue to red. The cross-section indicated by the dashed line is shown in red in **a**.

We determined the spatial coherence of the emitted light using a charge-coupled device beam profiler (Methods). Above threshold, directional beam emission with a small divergence angle (*θ*<1.5°) was observed normal to the sample surface for pumping along both the high-symmetry direction and 45° with respect to the high-symmetry direction (Fig. 1d). The lasing beam was extended slightly along the high-symmetry direction of the Au NP arrays from off-normal ASE^14^ (Supplementary Fig. 2; Supplementary Movie 1). Far-field patterns measured at different distances from the NP array showed negligible beam divergence (Supplementary Fig. 3). Photogenerated excitons from IR-140 have lifetimes of ∼900 ps^18^ (Supplementary Fig. 4a) that can be reduced in the presence of plasmonic NPs from strong local optical confinement and the Purcell effect^10^,^14^,^17^,^19^. We used time-correlated single-photon counting (TCSPC) to determine exciton dynamics below and above threshold at the lasing wavelength (*λ*_lasing_=865 nm for this device). Figure 2a indicates that below threshold (0.080 mJ cm^−2^), SE dominated with a single exponential decay time of 715 ps, close to the intrinsic lifetime (900 ps). Although there may be faster decay components, these were obscured by the large background from excess dye molecules in the nanolasing device unaffected by the near-field electromagnetic field confinement of the NPs (measured thickness of liquid gain was ∼100 μm). As the pump intensity increased and approached the threshold level (0.095 mJ cm^−2^), stimulated emission started to dominate at 865 nm, and the measured lifetime was reduced from 715 to 21 ps (Fig. 2a) as determined from a bi-exponential fit to the data deconvolved with the instrument response function (Supplementary Fig. 4a). Above threshold (0.144 mJ cm^−2^), the fast decay component was further reduced from 21 to 12 ps and was limited by the resolution of our TCSPC set-up (Methods). Due to the spatial distribution of the dye molecules in the vicinity of the Au NPs, uncoupled or weakly coupled molecules resulted in longer decay components (∼400 ps) even above threshold (Fig. 2a).

We simulated interactions between electromagnetic fields and the active gain medium using a semi-quantum framework based on a four-level gain system (Supplementary Fig. 5) and a time-domain approach developed previously^14^,^20^ (Methods; Supplementary Note 1). Briefly, macroscopic polarization equations for the absorption and emission transitions and rate equations for the gain medium, as well as Maxwell's equations were solved self-consistently. Time evolution of the inverted population Δ*N* between the state |2> and the state |1> was calculated to acquire decay lifetime information (Supplementary Fig. 6), and a reduction of lifetime with increased pump intensity was observed. The simulated lifetimes below, approaching, and above threshold were qualitatively consistent with our measured data. The analysis of gain and loss based on lifetimes (Supplementary Note 2) revealed that the gain was enhanced by the plasmonic cavity to overcome the loss and enable lasing action.

Above threshold, lasing signals clearly dominated SE at the operating wavelength; this trend was further confirmed by measuring the decay time of photons emitted at different wavelengths. We performed a wavelength-dependent decay time experiment where decay curves were measured from 860 to 880 nm with 1-nm steps and compiled to create a map (Fig. 2b, see Methods). An intense spot with ps-emission time resolution was observed around 865 nm, the lasing wavelength, whose lateral width was ca. 2 nm. This intense spot had a tail extending into the sub-ns regime that can be attributed to the excess dye molecules not interacting with the near-field of the Au NPs^18^. This observation was strikingly different from the IR-140-DMSO-only control sample (Supplementary Fig. 4b), where only SE with ns lifetimes was observed over the entire spectral range.

Besides the lasing action by lattice plasmons at the band edge, ASE due to propagating lattice plasmons can be observed at off-normal directions with a threshold higher than that of lasing^14^. We also confirmed this trend with liquid gain media. At 10° with *λ*_ASE_=877 nm, the ASE threshold (0.15 mJ cm^−2^) was higher than the lasing threshold (0.1 mJ cm^−2^). Approaching the lasing threshold (0.095 mJ cm^−2^), the ASE lifetime was reduced from 619 to 180 ps (Supplementary Fig. 7a), ca. one order of magnitude longer than the lifetime of the lasing mode. At pump intensities above the lasing threshold (0.143 mJ cm^−2^), the ASE lifetime decreased to 21 ps; above the ASE threshold (0.238 mJ cm^−2^), the ASE lifetime further decreased to 17 ps from the amplification of propagating lattice plasmons by stimulated emission. Compared with the highly directional lasing emission, ASE (Supplementary Fig. 7b) followed the (−1, 0) Rayleigh anomaly mode (Supplementary Fig. 2b). This phenomenon can be exploited for directional fluorescence enhancement^21^.

### Tuning lattice plasmon modes by changing refractive index

**Figure 3:**
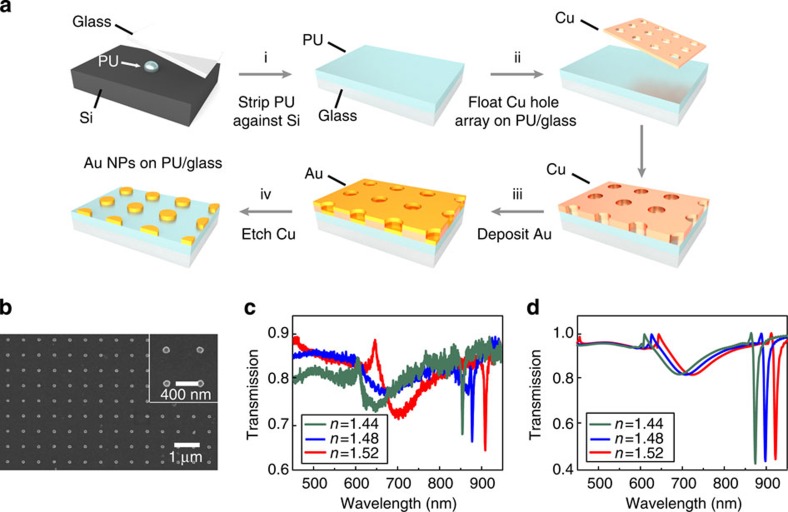
Au NP arrays with tunable lattice plasmon resonances by changing refractive index. (**a**) The fabrication technique consists of (i) producing substrates polyurethane (PU) on glass (denoted as PU/glass) with different refractive indices by stripping PU against Si; (ii) floating Cu hole arrays onto PU/glass; (iii) depositing Au; and (iv) etching Cu hole arrays. (**b**) Scanning electron microscope image of Au NPs on PU/glass. (**c**) Transmission experiments and (**d**) simulations of Au NPs in different dielectric environments (*n*=1.44, 1.48 and 1.52).

Lattice plasmon modes associated with the coupling between diffractive modes of the lattice and LSPs of individual metal NPs^22^ are sensitive to the size and spacing of the NPs^23^,^24^. However, exploiting these modes for real-time tunable plasmon lasing requires that the size and spacing of the NPs are fixed. Therefore, our approach focused on tailoring the refractive index environment of the superstrate and substrate, as lattice plasmons are optimized in a homogeneous dielectric environment^14^,^23^,^25^. Figure 3a summarizes our strategy to create plasmonic NP arrays on transparent substrates with different refractive indices. First, we prepared near-atomically flat polyurethane (PU) substrates by template-stripping PU against clean Si (100) wafers. We chose PU because this polymer is commercially available with a variety of refractive indices. Next, Cu nanohole arrays created by PEEL (Methods)^26^, a process combining photolithography, etching, electron-beam deposition and lift-off, were floated onto PU/glass substrates to form a physical deposition mask (Supplementary Fig. 8a). Then, we deposited 50-nm Au and etched the Cu hole array to create a square array of Au NPs (diameter *d*=120 nm, height *h*=50 nm) over cm^2^-areas. Besides PU substrates, this approach also enabled large-scale patterning of metal NP arrays on both rigid (glass) and flexible (polymers) substrates (Supplementary Fig. 9).

Figure 3b shows a scanning electron microscopy image of Au NP arrays on PU/glass produced via the process described in Fig. 3a. We selected the particle dimensions (*d*=120 nm, *h*=50 nm) to achieve lattice plasmon resonances with high-quality factors (*Q*), given the lattice spacing of the Cu hole arrays (*a*_0_=600 nm). We found that the uniformity of the NPs was critical to produce extremely narrow resonances, although some level of structural dispersion can be tolerated based on the collective nature of the lattice plasmon resonance^23^,^25^. Figure 3c indicates that lattice plasmon modes with exceptionally narrow FWHM (4 nm) and high *Q* (∼230) can be achieved using Cu hole arrays as the deposition mask. These *Q* values are ca. 20 times higher than that of a LSP from a single metal NP of the same size (Q<10, calculated from the broad resonance at ca. 650–700 nm). Lattice plasmon modes are dispersive and follow the (0, ±1) Rayleigh anomaly line under transverse magnetic polarized light^14^ (Supplementary Fig. 8b). At the band edge (*k*=0), Au NP arrays supported in-phase charge oscillations and strong near-field enhancement 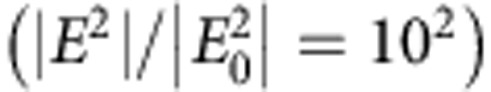 (Supplementary Fig. 8c).

By patterning Au NP arrays of the same dimensions on PU substrates with different refractive indices (*n*=1.44–1.52), we found that lattice plasmons could be tuned over a wide wavelength range (858–913 nm) as a linear function of *n*. Transmission spectra of Au NPs in different *n* environments were calculated using a finite-difference time domain (FDTD) (Methods). Excellent agreement with experiments was observed (Fig. 3d). We thus demonstrated that lattice plasmon resonances could be tuned by altering the dielectric environment without needing to change NP size and lattice spacing. Importantly, this strategy for tuning lattice plasmons combined with liquid gain materials makes possible the ability to modulate the lattice plasmon lasing wavelength in real time.

### Static wavelength tuning of lattice plasmon lasers

To demonstrate lasing wavelength tunability, we kept the geometric parameters of the Au NP arrays constant (*a*_0_=600 nm, *d*=120 nm, *h*=50 nm) and changed the dielectric environment of the gain. We selected a range of different *n* (1.42, 1.44, 1.46, 1.48 and 1.50) in which the *Q* of the lattice plasmon modes would still be high and that would produce resonances within the IR-140 PL bandwidth. For substrates with a given *n*, we used template-stripped PU/glass. For liquid gain superstrates with matching *n*, we first dissolved IR-140 in ethylene glycol (EG, *n*=1.43)^27^, DMSO (*n*=1.48) and benzyl alcohol (BA, *n*=1.54), organic solvents reported to show high solubility (ref. 28). These solvents were then mixed to match the index of the substrates to produce a homogeneous dielectric environment for the Au NPs (Methods). We checked the PL of IR-140 in these solvents as controls (Supplementary Fig. 10a) and found that PL varied slightly; IR-140 dissolved in DMSO had a stronger and more stable emission compared with IR-140 in BA or EG. The intrinsic absorption of these solvents (and mixtures of them) is negligible in the wavelength range 500–1,000 nm, and the output power of the nanolaser will not be affected.

**Figure 4:**
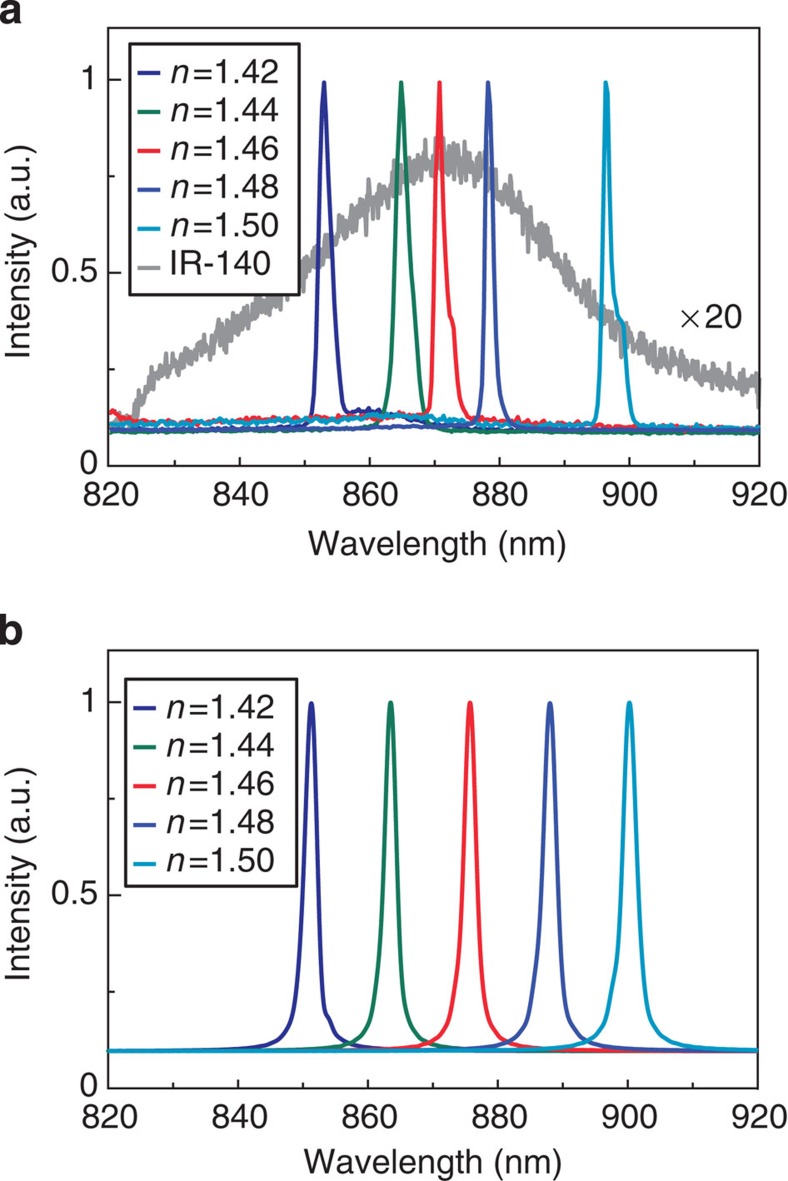
Lasing emission at different wavelengths. (**a**) Experiments and (**b**) simulations of lasing emission from Au NP arrays embedded in different index environments from *n*=1.42 to *n*=1.50 (gain concentration: 1 mM; pump intensity: 0.188 mJ cm^−2^). The emission intensities were normalized.

Figure 4a highlights that lattice plasmon lasing can be tuned over the entire gain bandwidth of the dye. Optically pumped at 800 nm, nanolaser devices showed lasing with wavelengths from 853 to 896 nm. Using a single type of dye molecule, the tunability range was Δ*λ*_lasing_=43 nm. In contrast, to achieve similar tuning ranges, DFB lasers need to use either different grating periods or different thicknesses of the dye layer^3^,^4^. At the same pump intensity above threshold (0.188 mJ cm^−2^), the amplitude of the lasing signal depended on emission wavelength. In particular, the lasing signal at 871 nm had the strongest emission due to better overlap of the plasmonic mode with the emission of IR-140, as well as the stronger PL of IR-140 in DMSO (Supplementary Fig. 10b). Figure 4b shows that simulated lasing spectra using our semi-quantum framework^14^,^20^ matched well with experiment. In calculations, we considered a dye emission profile centred at 870 nm with a bandwidth of 50 nm, and found that the amplitude of the lasing signals indeed followed the PL profile (Supplementary Fig. 10c). Small discrepancies between simulations and experiments of the lasing wavelengths are most likely due to mismatch between the nominal and the empirical values of the refractive index.

### Dynamic real-time tuning of lattice plasmon lasers

**Figure 5:**
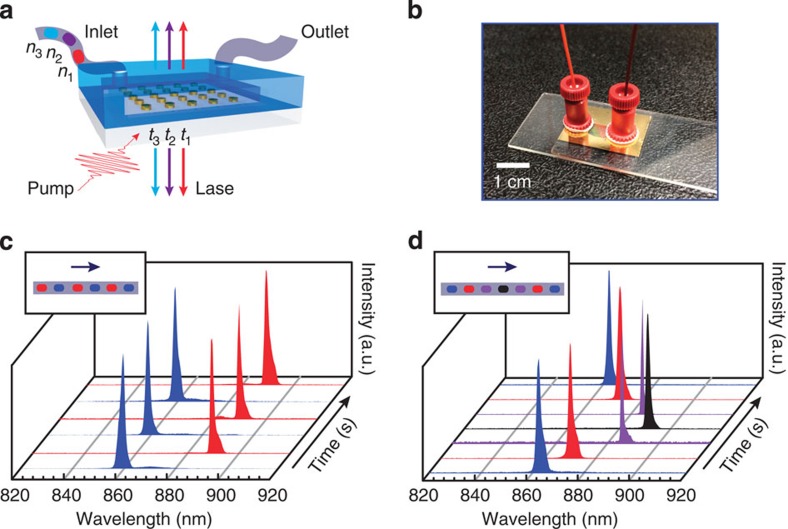
Lasing emissions from Au NP arrays tuned in real time. (**a**) Scheme of the dynamic laser, (**b**) photograph of the device, (**c**) switching lasing wavelengths and (**d**) shifting lasing wavelengths. In **c**, IR-140 dye molecules were dissolved in DMSO (blue) and BA (red). In **d**, IR-140 dye molecules were dissolved in DMSO (blue), DMSO: BA=2:1 (red), DMSO: BA=1:2 (purple) and BA (black). DMSO: dimethyl sulfoxide, BA: benzyl alcohol. The emission intensities were normalized.

One advantage of lattice plasmons is that they can tolerate a certain amount of refractive index contrast (mismatch) between the superstrate and substrate^29^. We determined that this contrast Δ*n* could be as large as 0.05 in experiments with Au NP arrays still showing high-quality resonances (Supplementary Fig. 11). Thus, besides static tunability of the nanolaser demonstrated in Fig. 4, this system can now be integrated for testing and realizing a dynamically tunable plasmon-based laser. We exploited the large-area patterns of Au NP arrays (∼cm^2^) and integrated them into a microfluidic device where the liquid gain with different *n* could be introduced into the system in real time (Fig. 5a). Figure 5b represents one of our nanolasing devices consisting of Au NP arrays and a microfluidic channel fabricated by sandwiching a silicone sheet (thickness=250 μm) with a 5-mm-wide and 15-mm-long slit in the centre between the Au NP substrate (silica) and a glass microscope slide (Methods).

Figure 5c shows that the lasing emission can be switched between two wavelengths (*λ*_1_=859 nm and *λ*_2_=890 nm) by alternating plugs of liquid dye between IR-140-DMSO and IR-140-BA across Au NP arrays on fused silica. Dynamic lasing emission in a non-homogeneous environment was not as stable as in a static environment with a homogenous index (Fig. 4). However, a distinct advantage of a dynamic system is that the gain materials can be constantly replenished. Continuous flow of the liquid gain over the Au NPs can refresh the dye molecules that are exposed to the excitation beam, as well as extend the lifetime stability of the device by avoiding photo-oxidation and photobleaching of dye molecules^30^. We noticed that the linewidth and stability of the lasing signal also depended on the quality of the microfluidic channel (Supplementary Movie 2). Not surprisingly, the lasing devices showed much narrower and stronger emission when the substrate and the superstrate were closer in index, such as for glass (*n*=1.52) and IR-140-BA (*n*=1.54) (Supplementary Movie 3).

The speed of switching the lasing wavelength could be controlled by adjusting the volume and flow rate of each plug of liquid dye. Our switching experiments were on the order of seconds, but this rate could easily be improved (for example, ms) in an optimized set-up. Significantly, Fig. 5d shows that the lasing wavelength can be continuously shifted to longer wavelengths and then back again to shorter wavelengths with no hysteresis (Supplementary Movie 4). IR-140-DMSO (*n*=1.48), IR-140-BA (*n*=1.54) and their solutions in 2:1 (*n*=1.50) and 1:2 (*n*=1.52) volume ratios were used as liquid gain plugs and were injected with increasing *n* from IR-140-DMSO to IR-140-BA and then with decreasing *n* back to IR-140-DMSO. Thus, the lasing wavelength was tuned from 862 to 891 nm and then back to 862 nm. Dynamic control of the lasing emission could open opportunities for applications such as real-time control of enhanced light–matter interactions on the nanoscale.

## Discussion

In summary, we designed and realized a plasmon nanolaser whose emission properties can be modulated in real time. Liquid gain with different refractive indices were able to tune the lattice plasmon resonance, and hence lasing wavelength, over the entire bandwidth of the dye. Moreover, we demonstrated that the lifetime of the gain was significantly reduced above the lasing threshold, in agreement with theory. Dynamic control of the far-field lasing emission and the nanolocalized lasing fields offer prospects for enhancing light–matter interactions on the nanoscale for real-time monitoring and detection. This system offers a diverse range of possibilities for both ultra-narrow lattice plasmons and ultrasensitive sensing applications, as well as for tunable nanolasers that can be integrated in lab-on-a-chip devices.

## Methods

### Fabrication of Cu hole arrays as deposition masks

A poly(dimethylsiloxane) mask with square lattice spacing *a*_0_=600 nm was used in phase-shifting photolithography^31^ to produce photoresist posts with diameter *d*=120 nm on Si (100) wafers. After depositing a thin layer of Ti (10 nm) and lift-off photoresist posts, we used deep reactive ion etching to create cylindrical pits beneath the circular Ti holes (diameter ∼120 nm and depth ∼150 nm) into the Si. Cu nanohole arrays were then produced by depositing 100-nm Cu and etching of the Ti sacrificial layer. Cu nanohole arrays were floated onto substrates as deposition masks and then could be removed by Cr etchant.

### Lasing device fabrication

The lasing device consists of Au NP arrays patterned on substrates with different *n* and IR-140 (Sigma-Aldrich) dissolved in different solvents as the gain media. A droplet of the IR-140-solvent liquid was deposited onto Au NP arrays and sandwiched between this substrate and another piece of cover glass (the gain layer was ∼100 μm). The solvents for dissolving the dye were EG, DMSO and BA. According to the refractive index of the substrate, we chose one or mixed two solvents to obtain close index values. (Note: the refractive index at the sodium D line 589.3 nm and 20 °C is denoted as nD20. The nD20 of these solvents are 1.438, 1.477 and 1.538, respectively. Because the solvents have a dispersive refractive index, the indices at ∼900 nm are smaller than those measured at 589.3 nm.) Specifically, we used EG for both substrate *n*=1.42 and *n*=1.44, EG: DMSO=1:1 for substrate *n*=1.46, DMSO for substrate *n*=1.48 and BA for substrate *n*=1.50.

For real-time lasing measurements, a fluidic channel was built to deliver the IR-140-liquid over Au NP arrays. The microfluidic channel was fabricated by sandwiching a silicone sheet (Grace Bio Laboratories, thickness 250 μm), with a 5-mm-wide and 15-mm-long slit cut in the centre, between the Au NP arrays and a glass microscope slide. Inlet and outlet holes were drilled through the glass slide providing openings for tubing connections (N-126S Upchurch Scientific NanoPort Assembly and 1569 PEEK Tubing). A syringe pump (kdScientific) connected to an injection valve (Rheodyne 9725) was used to introduce the IR-140-liquid with a constant flow rate of 30 μl min^−1^.

### Lasing measurements

A mode-locked Ti:sapphire laser with a regenerative amplifier (800 nm wavelength, 1 kHz repetition rate and 90 fs pulse width) was used to optically pump the device at an incident angle of 45°. The emission was collected at different detection angles and then coupled into a bundled optical fibre connected to a compact spectrometer (USB 2000, Ocean Optics, 0.3 nm resolution). The laser beam was polarized along the high-symmetry axis of the square lattice. The spatial pattern and divergence angle of the lasing beam were analysed using a high-resolution charge-coupled device beam profiler (LBR-HR, Newport, 1.4 Megapixel) placed at different distances normal to the sample surface.

### Time-correlated single-photon counting

For fluorescence lifetime measurements, a mode-locked Ti:sapphire laser (Coherent Libra, 1 kHz repetition rate, 100 fs pulse width) at 800 nm, 45 degree incident angle was used for excitation. Fluorescence of IR-140 was collected by a × 5 and 34-mm working distance Mitutoyo objective. The excitation laser and fluorescence signal were filtered by 800 nm bandpass and 820 nm longpass filters (Semrock), respectively. The filtered fluorescence was then coupled to a single-mode fibre and guided to the entrance slit of a spectrometer (Princeton Instrument SP2500). After being dispersed by a grating, the fluorescence was diverted to a second exit port of the spectrometer (∼1.5 nm bandwidth) and collected by a fast-timing avalanche photodiode (APD-PDM, Micro Photon Device). A TCSPC module (PicoHarp 300) with a time bin of 4 ps was used to analyse the number of photons as a function of time, when they arrived at the photodiode. Final lifetimes were obtained from fits to the data deconvolved with the instrument response function^32^.

### Numerical simulations of lasing emission

The active gain medium (dye molecules) was modelled as a four-level quantum system, and the interactions between the dye molecules and the electromagnetic fields were simulated by solving the net macroscopic polarization equation for the absorption and emission transitions, the rate equations and the Maxwell's equations using an in-house three-dimensional FDTD code^14^,^20^ (Supplementary Note 1).

### Finite-difference time-domain simulations

FDTD calculations based on commercial software (FDTD solution, Lumerical Inc., Vancouver, Canada) were used to simulate the linear properties of Au NPs. The optical constants of Au were taken from Johnson and Christy^33^ (450–950 nm). A uniform mesh size of 2 nm (*x*, *y* and *z* directions) was used. The size of Au NPs was diameter *d*=120 nm, spacing *a*_0_=600 nm and height *h*=50 nm.

## Author contributions

A.Y. and T.W.O. conceived the idea of a real-time, tunable lattice plasmon laser based on a liquid dye and Au nanocavity arrays. A.Y. fabricated the devices, carried out the optical measurements and performed FDTD numerical simulations of the passive optical responses of the devices. T.B.H. carried out lifetime measurements using time-correlated single-photon counting. M.D. simulated the active optical responses of the device. A.Y. and C.D. carried out lasing measurements. T.W.O., M.H.M. and G.C.S. guided experimental and theoretical investigations. A.Y. and T.W.O. analysed the data and wrote the manuscript. All authors commented on and revised the manuscript.

## Additional information

**How to cite this article:** Yang, A. *et al*, Real-time tunable lasing from plasmonic nanocavity arrays. *Nat. Commun*, 6:6939 doi: 10.1038/ncomms7939 (2015).

## Supplementary Material

Supplementary Figures, Notes and References Supplementary Figures 1-11, Supplementary Notes 1-2 and Supplementary References

Supplementary Movie 1 Amplified spontaneous emission at off-normal angles. The incident pump laser was horizontally polarized. The detector was moved slightly (ca. 1°) vertically (horizontally for the second half of the movie) and then brought back. An ASE signal emerged out of the lasing mode and shifted when the detector moved (from ca. 870 nm to ca. 890 nm). In contrast, the lasing mode stayed at ~ 865 nm without shifting.

Supplementary Movie 2 A demonstration of both switching and shifting. In this test, the liquid dye injected in the channel as IR-140-BA, IR-140-DMSO, and IR-140-BA used DI water as the flow medium. Therefore, the first and the last plugs of IR-140-BA were partially diluted by water and had lower refractive index than IR-140-BA. The lasing signal showed up first at ca. 878 nm (IR-140-BA-water) and stabilized at ca. *λ*_1_ = 883 nm (IR-140-BA). Then, the lasing emission switched to *λ*_2_ = 865 nm when IR-140-DMSO was introduced. The lasing quickly switched to *λ*_1_ = 883 nm again (IR-140-BA) and gradually shifted towards 865 nm due to the partial dilution by DI water.

Supplementary Movie 3 Dynamic laser: switching between two lasing wavelengths. The lasing wavelength was switched between *λ*_1_ = 865 nm and *λ*_2_ = 888 nm by alternating IR-140-DMSO and IR-140-BA as gain for Au NPs on glass substrate. The signal at *λ*_2_ = 888 nm was stronger than the signal at *λ*_1_ = 865 nm because the index of IR-140-BA (*n* = 1.54) matched more closely with the glass substrate (*n* = 1.52).

Supplementary Movie 4 Dynamic laser: continuous shifting of the lasing wavelength. In this demonstration, IR-140-DMSO and IR-140-BA were mixed with ratios 2:1 and 1:2 to obtain another two liquid gain media. These four liquids were injected with increasing n from IR-140-DMSO (*λ*_lasing_ = 865) to IR-140-BA (*λ*_lasing_ = 885 nm) and then with decreasing n back to IR-140-DMSO over Au NP arrays on fused silica. Because the fused silica has an index *n* = 1.48, the lasing signal was strongest at shorter wavelengths and weakest at longer wavelengths.
