# Supplementary material for: Real-time tunable lasing from plasmonic nanocavity arrays
Source: Nat Commun. 2015 Apr 20;6:6939. doi: 10.1038/ncomms7939 (PMC4411284; doi:10.1038/ncomms7939)
Supplement: Supplementary Figures, Notes and References — Supplementary Figures 1-11, Supplementary Notes 1-2 and Supplementary References [file ncomms7939-s1.pdf]

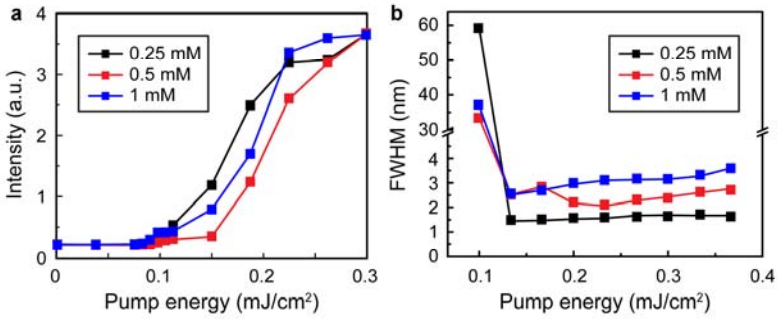

Supplementary Figure 1. (a) Threshold and **(b)** line-width of the lasing emission versus pump intensity. Because the IR-140 dye is dissolved in organic solvents, we can determine whether there are any concentration-dependent effects of the liquid gain on the lasing signal. For this experiment, we used Au nanoparticles (NPs) on a fused silica substrate with IR-140-DMSO as superstrate. The threshold level was similar (~ 0.1 mJ/cm^2) when the concentration increased from 0.25 mM to 1 mM, which suggests that the primary losses in the system were from the plasmonic NPs. In contrast, the lasing line-width narrowed from ~ 3 nm to ~1.5 nm as the concentration decreased.

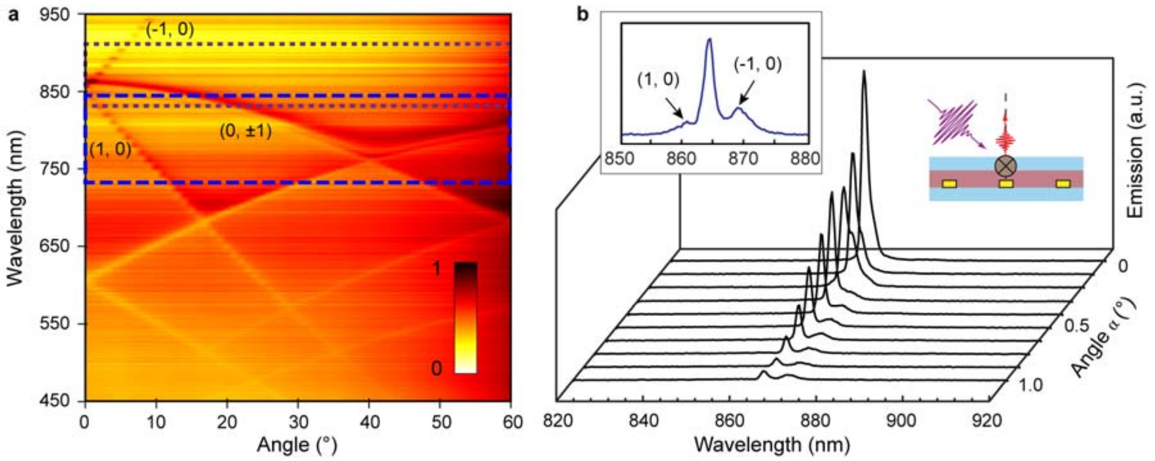

Supplementary Figure 2. Off-angle ASE detected at small angles. (a) Band structure of Au NPs in a homogeneous dielectric environment (n = 1.46). Au NP arrays under unpolarized light support three dispersive lattice plasmon bands following the (-1, 0), (0, ±1), and (1, 0) Rayleigh anomaly modes. The blue and purple rectangular boxes indicate the absorption and emission bands of IR-140 in DMSO. The peak absorption of IR-140 is ca. 820 nm with a FWHM of ~ 80 nm, and the peak emission is ca. 870 nm with a FWHM of ~ 50 nm. **(b)** Off-angle ASE signals detected perpendicular to the incident plane, inset: ASE signals following (± 1, 0). The ASE signals shift to longer wavelengths with the increased angle. At the band edge, all modes are degenerate and only the lasing signal was detected. When the angle was increased to ca. 1°, the emission of the dye molecules coupled into the lattice plasmon band (-1, 0) resulted in ASE away from the lasing and also reduced the lasing intensity. When the angle further increased (> ca. 15°), both the lasing and ASE signals disappeared. The emission that coupled into the band (+1, 0) only appeared at a very small angle.

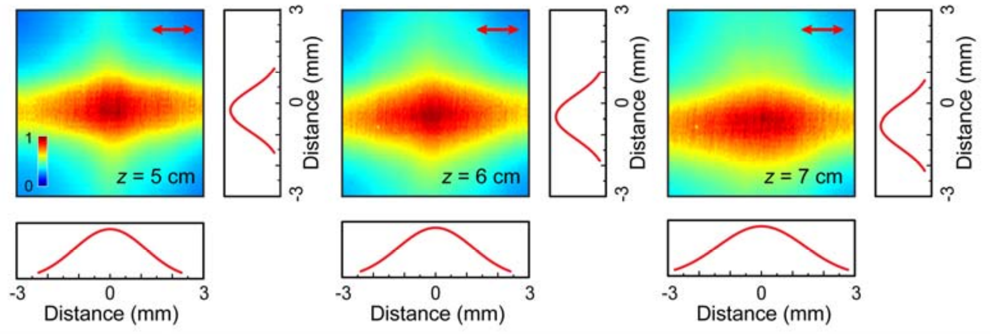

Supplementary Figure 3. Far-field emission pattern at different distances. The beam exhibited Gaussian distributions along both *x* and *y* directions. The arrows indicate polarization directions. The samples were Au NPs on glass substrate covered with IR-140 in benzyl alcohol. A charge-coupled device (CCD) beam profiler was used to map the far-field pattern of the emitted lasing signal at different distances from 5 cm to 7 cm to determine spatial coherence. The beam only slightly expanded over the distances measured, indicating high directionality of the emission. The beam extended along the high-symmetry grating direction due to off-angle ASE.

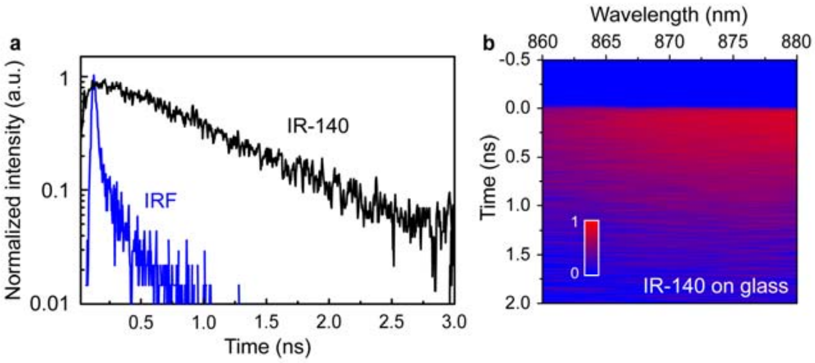

Supplementary Figure 4. Time-correlated single photon counting (TCSPC). (a) IRF of TCSPC set-up (~ 30 ps) and lifetime of IR-140 dye molecules on glass (~ 0.9 ns). **(b)** Lifetime of IR-140 on glass as a function of the emission wavelength collected normal to the surface above lasing threshold at 0.173 mJ/cm^2. TCSPC was used to measure the decay time of IR-140 dye molecules at various pump intensities. The IRF exhibited a ~ 30 ps decay while the IR-140 dissolved in DMSO had a lifetime of ~ 0.9 ns on a glass substrate. For lifetimes shorter than the IRF, the system will return a curve similar to the IRF. To extract the lifetime, we fit the data and deconvolved with the measured IRF. In order to measure the decay time at a given wavelength, the signal was first dispersed by a spectrometer grating and filtered by an exit slit. The wavelength-dependent decay time data performed on the IR-140-DMSO-only as control showed only spontaneous emission 860 nm to 880 nm with a ~ 0.9 ns lifetime.

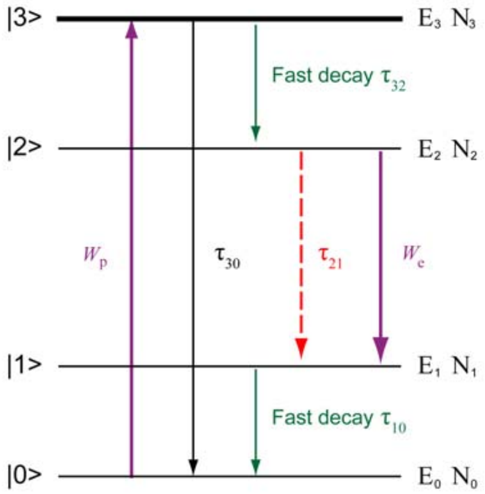

Supplementary Figure 5. Four-level system of the dye molecules. The pumping rate is $W_p = \vec{E} \cdot d\vec{P}_{30}/dt$, the lifetime of the spontaneous emission τ_{21} is a function of the distance from the NPs according to the Purcell effect. Fast non-radiative decay rates are assumed between states |3> and |2> and between states |1> and |0> (τ_{32}= τ_{10}= 10 fs). An inverted population is achieved between the states |2> and the state |1> with a stimulated emission rate $W_e = \vec{E} \cdot d\vec{P}_{21}/dt$.

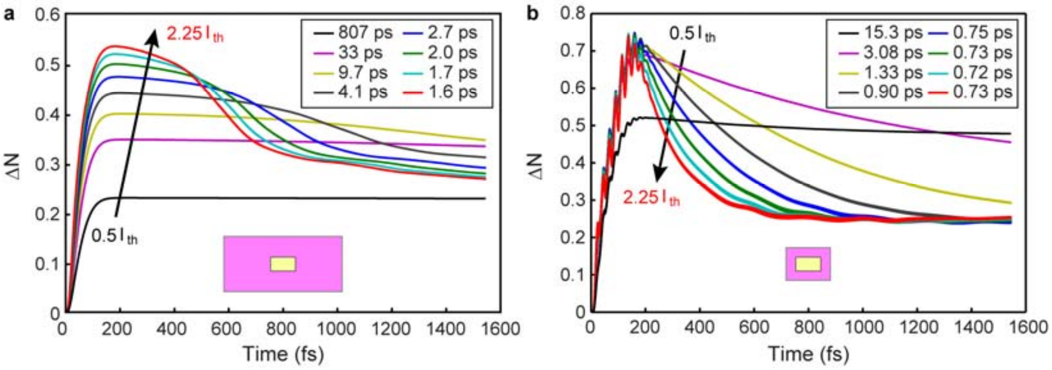

Supplementary Figure 6. Lifetime extracted from the inverted population ΔN. **(a)** ΔN was averaged from the whole volume of the gain layer and **(b)** ΔN was averaged within 25 nm to the surface of the Au NPs.

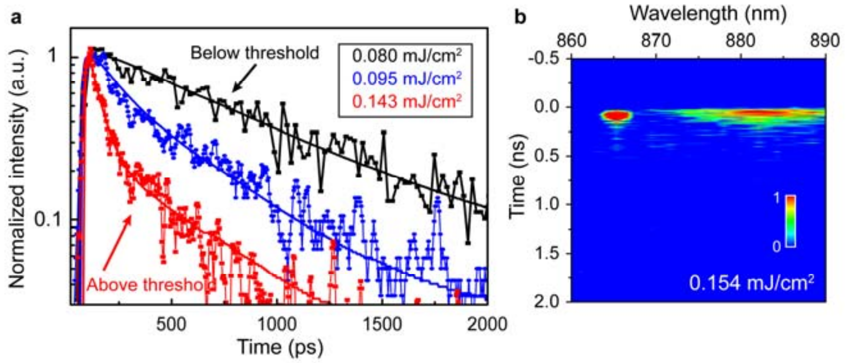

Supplementary Figure 7. Lifetime of amplified spontaneous emission. (a) Decay times of emission at 877 nm measured at pump intensities below and above lasing threshold (0.1 mJ/cm^2) showing a reduction in lifetime at higher pump intensities. The solid lines show fits to the data deconvolved with the IRF. **(b)** Lifetime as a function of the emission wavelength collected at a 10° angle and above ASE threshold at 0.154 mJ/cm^2. Amplified spontaneous emission (ASE) was observed at off normal angles with a threshold of 0.15 mJ/cm^2. The ASE lifetime at 877 nm (collection angle: 10°) was measured using TCSPC for comparison with the lifetime of the lasing mode at 865 nm (Figure 2). The lifetime decreased from 619 ps to 180 ps when the pump intensity (0.095 mJ/cm^2) approached the lasing threshold value (0.1 mJ/cm^2), which was one order of magnitude longer than the lifetime of the lasing mode at the same pump intensity. When the pump intensity was above lasing threshold, at 0.143 mJ/cm^2, and above ASE threshold, at 0.238 mJ/cm^2, for example, the ASE lifetime was further reduced to 21 ps and 17 ps. We also measured the decay time of dye molecule emission from 860 nm to 890 nm at a pump intensity of 0.154 mJ/cm^2 to study the wavelength-dependence of the lifetime above threshold. A broad emission peak from ca. 870 nm to 890 nm exhibited a reduction in lifetime (~ 20-250 ps). Note: the lasing mode at 865 nm was also resolved in the scan because our objective (NA = 0.14) was located at 34 mm from the surface and had a large field view (~ 17°); even with the small lasing beam divergence (~1.5°), the objective was still able to collect lasing signal due to the spatial distribution of the lasing emission from the ~ 0.2 cm^2 excitation area.

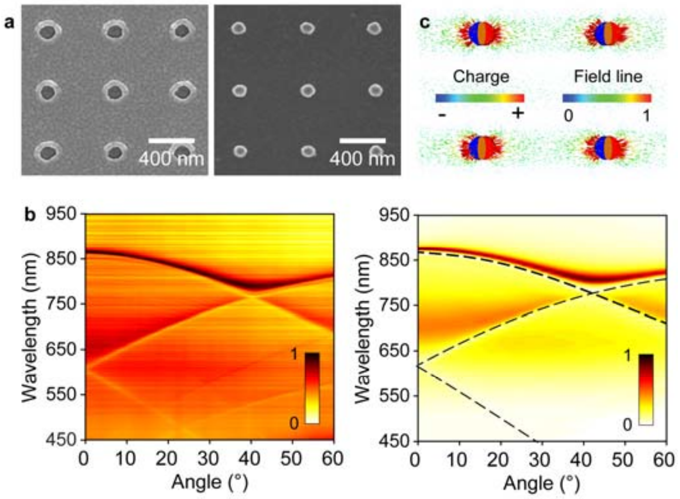

Supplementary Figure 8. Lattice plasmon resonance. (a) SEM image of the Cu hole arrays and Au NPs on PU/glass (a_0 = 600 nm, d = 120 nm, h = 50 nm). **(b)** Measured and simulated dispersion diagram for Au NPs (a_0 = 600 nm, d = 120 nm, h = 50 nm) for index n = 1.44 under TM polarization. In the simulation, the thick (thin) dashed lines represent the first (second) order diffraction modes. The lattice plasmon resonance is strongest in a homogeneous environment and is sensitive to the refractive indices of both the superstrate and substrate. For n = 1.44, the Rayleigh anomaly line starts around 600 nm × 1.44 = 864 nm. The diameter of the NPs needs to be selected such that the plasmon band matches the chosen lattice spacing (multiplied by n). **(c)** Simulated charge distribution around Au NPs at the band-edge. Au NP arrays support dispersive lattice plasmons that follow the Rayleigh anomaly line from diffractive coupling of localized surface plasmons (LSPs) of each individual NP in the array.[1] At the band edge, NPs support an extremely narrow geometrical resonance and intense localized electromagnetic fields. On resonance, all NPs exhibit an in-phase dipolar charge distribution.

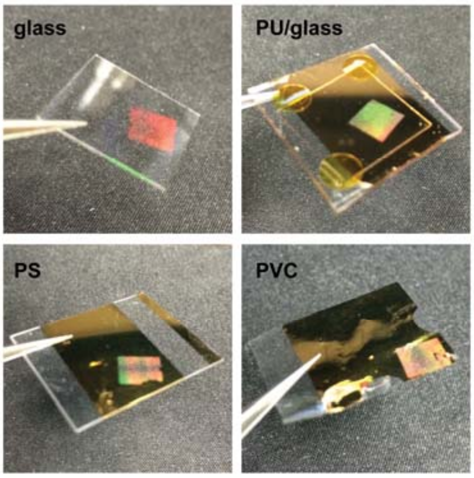

Supplementary Figure 9. Patterning Au NP arrays on various substrates. The fabrication technique described in Figure 3 enables the patterning of metal NP arrays on various substrates from stiff glasses to flexible polymers. Briefly, Cu nanohole arrays fabricated by the PEEL method[2] were used as physical masks for the deposition of NPs on substrates. Cu nanohole arrays were then removed using Cr etchant to leave NP arrays on the substrates. Cr etchant is compatible with most of the glasses and polymers and can be rinsed away without adversely affecting the substrates. Glass, polyurethane (PU), polystyrene (PS) and poly(vinyl chloride) (PVC) were used as examples to show the versatility of this technique. This technique can find applications in, for example, patterning plasmonic structures and metamaterials onto flexible electronic devices.

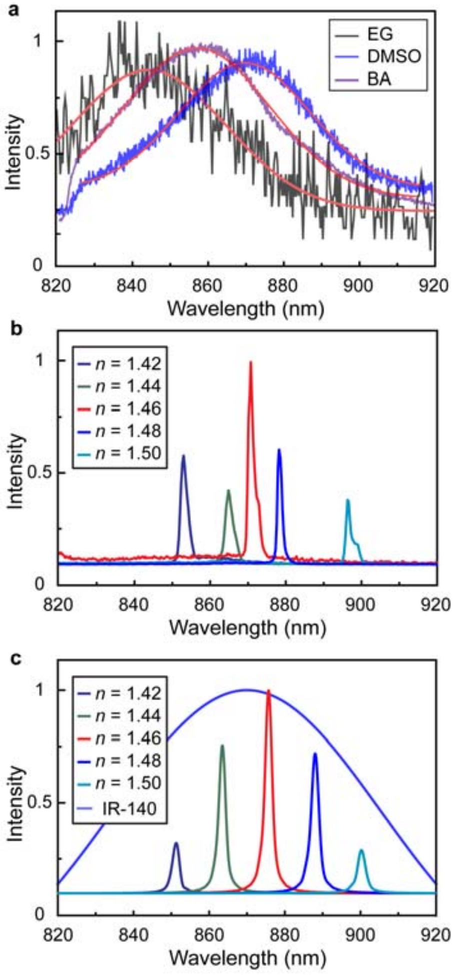

Supplementary Figure 10. Emission of IR-140 dye molecules and relative lasing signals. (a) Photoluminescence of IR-140 in different solvents. EG: ethylene glycol, DMSO: dimethyl sulfoxide and BA: benzyl alcohol. **(b)** Experimental and **(c)** calculated lasing emission at different wavelengths. The intensities were normalized to the strongest signal. The center wavelength and emission bandwidth were 844.3 nm and 48.7 nm for EG; 870.0 nm and 41.5 nm for DMSO; 856.8 nm and 46.1 nm for BA. IR-140 in DMSO exhibited the best performance (i.e., strong and stable emission), with BA second and EG third. At the same pump intensity above threshold (0.188 mJ/cm^2), the amplitude of the lasing signal depended on the emission wavelength, where: (1) IR-140-EG was used for substrates with n = 1.42 and n = 1.44. The signal for n = 1.42 was stronger than n = 1.44 due to the better overlap between the plasmonic mode and the dye emission; (2) IR-140-EG: DMSO (1:1) was used for substrate with n = 1.46.

This sample showed the strongest signal from the strong emission of IR-140 in DMSO as well as good match of the plasmon mode with the dye emission; (3) IR-140-DMSO was used for substrate n = 1.48, and the signal was also strong; and (4) IR-140-BA was used for substrate n =1.50, and due to mismatch between the plasmonic mode and the dye emission, the signal was relatively weaker. In calculations, we considered a dye emission profile centered at 870 nm with a bandwidth of 50 nm and found that the amplitude of the lasing signals indeed followed the photoluminescence profile.

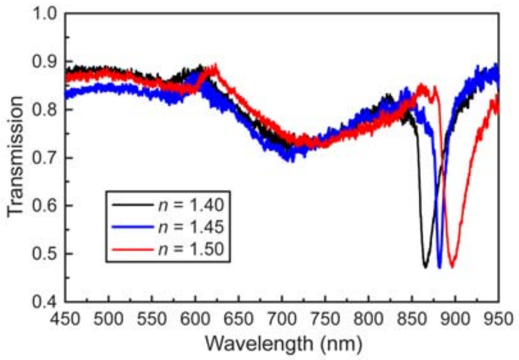

Supplementary Figure 11. Lattice plasmon resonance tolerates index contrast. Fused silica substrates had n = 1.46 and superstrates varied from 1.40 to 1.50. Lattice plasmon resonances are strongest and narrowest in a homogeneous environment but can tolerate a certain amount of index contrast (mismatch) between substrate and superstrate. We experimentally determined the quality of the plasmon mode using fused silica (n = 1.46) as the substrate with Au NPs (a_0 = 600 nm, d = 120 nm, h = 50 nm). The narrowest lattice plasmon resonance was when the superstrate had an index n of 1.45 (FWHM = 10 nm) (blue curve). When the superstrate had n = 1.40 or n = 1.50, the resonance broadened to FWHM = 27 nm and to FWHM = 26 nm, respectively. Note that these lattice plasmon resonances still supported high quality factors (Q = 30-40) compared to a single Au NP (Q <10).

Supplementary Note 1: Numerical Simulations of Lasing Emission

We used a semi quantum framework reported recently[3,4] to simulate the interaction between the electromagnetic fields and gain medium. The dye molecules (active medium) were described as a four-level system (Supplementary Figure 5) and population inversion was established between the state |2> and the state |1>. The molecular polarization resulting from spontaneous and stimulated transitions was calculated according to the following equation:[5]

$$\frac{d^2\vec{P}_{i,j}}{dt^2} + \Delta\omega_{i,j}\frac{d\vec{P}_{i,j}}{dt} + \omega_{i,j}^2\vec{P}_{i,j} = \kappa_{(i,j)}\,\Delta N_{i,j}(t)\vec{E}(t) \qquad \text{(Supplementary Equation 1)}$$

In Supplementary Equation 1, $\Delta\omega_{i,j}$ and $\omega_{i,j}$ are the bandwidth and frequency of the transition between states i and j (where this equation applies to (i, j) = (3, 0) and (2, 1)), $\kappa_{i,j} = 6\pi\epsilon_o c^3/(\omega_{i,j}^2 \cdot \tau_{i,j})$, and $\tau_{i,j}$ is the lifetime of the spontaneous emission. $\Delta N_{i,j}(t)$ is the population density difference between the two energy states of interest, $\vec{E}(t)$ is the total electromagnetic field calculated classically by solving Maxwell equations:

$$\nabla \times \vec{E}(t) = -\mu_0\frac{\partial\vec{H}(t)}{dt};\ \nabla \times \vec{H}(t) = \epsilon \cdot \frac{\partial\vec{E}(t)}{\partial t} + \frac{\partial\left(\vec{P}_{30}(t) + \vec{P}_{21}(t)\right)}{\partial t} \qquad \text{(Supplementary Equation 2)}$$

In these equations, we coupled the molecular polarizations $\vec{P}_{30}$ and $\vec{P}_{21}$ to the electromagnetic field and neglected the polarizations from other transitions because they were assumed to be very fast (10 fs). The time evolution of the populations N_i, and the population differences $\Delta N_{i,j}(t)$ were calculated from the rate equations:

$$\frac{dN_3(t)}{dt} = -\frac{N_3(t)}{\tau_{32}} - \frac{N_3(t)}{\tau_{30}} + \frac{1}{\hbar\omega_{30}}\vec{E}(t) \cdot \frac{d\vec{P}_{30}(t)}{dt} \qquad \text{(Supplementary Equation 3)}$$

$$\frac{dN_2(t)}{dt} = +\frac{N_3(t)}{\tau_{32}} - \frac{N_2(t)}{\tau_{21}} + \frac{1}{\hbar\omega_{21}}\vec{E} \cdot \frac{d\vec{P}_{21}(t)}{dt} \qquad \text{(Supplementary Equation 4)}$$

$$\frac{dN_1(t)}{dt} = +\frac{N_2(t)}{\tau_{21}} - \frac{N_1(t)}{\tau_{10}} - \frac{1}{\hbar\omega_{21}}\vec{E}(t) \cdot \frac{d\vec{P}_{21}(t)}{dt} \quad \text{(Supplementary Equation 5)}$$

$$\frac{dN_0(t)}{dt} = +\frac{N_1(t)}{\tau_{10}} + \frac{N_3(t)}{\tau_{30}} - \frac{1}{\hbar\omega_{30}}\vec{E}(t) \cdot \frac{d\vec{P}_{30}(t)}{dt} \quad \text{(Supplementary Equation 6)}$$

The time evolution of each quantum state density is governed by spontaneous decay processes $N_i/(\tau_{i,j})$ and stimulated processes ($\vec{E}(t) \cdot d\vec{P}_{i,j}/dt$). The field involved in Supplementary Equations 3-6 is the total field and accounts for the effects of any local optical intensity (e.g., plasmon enhanced near field) on the dynamics of the population densities.

The approach based on Supplementary Equations 1-6 is self-consistent in fields and populations. We also included the modification to the spontaneous decay rate arising from the Purcell factor using an approach described previously.[4] This leads to lifetimes that are a function of the spatial position of molecules in the cell. Overall, the formalism allows us to calculate the temporal and spatial profile of the population inversion as well as non-linear effects (saturation, gain depletion).

The finite-difference time-domain technique was used to solve the coupled equations (Supplementary Equations 1-6). Periodic boundaries conditions were applied in the *x* and *y* directions and an absorbing boundary condition based on the UPML (uniaxial perfect matched layer) technique was applied in the *z* direction. In our simulations, we assumed that all molecules were in the ground state at $t = 0$ ($N_3 = N_2 = N_1 = 0$). The dye parameters are: (1) absorption transition: λ_a = 800 nm and $\Delta\lambda_a$ = 50 nm; (2) emission transition: λ_e = 870 nm and $\Delta\lambda_e$ = 50 nm; (3) concentration of the dye: 1 mM; (4) lifetimes: τ_{30} = 1 ns, $\tau_{32} = \tau_{10}$ = 10 fs, $1/\tau_{21} = pf(x,y,z) \cdot 1/\tau_{21\_0}$ where τ_{21_0} = 1 ns in the free space, and $pf(x,y,z)$ is the Purcell factor calculated classically[4] as a function of spatial coordinate around the Au NPs.

The decay lifetime information was acquired by calculating the time evolution of the inverted population ΔN between the state |2> and the state |1>. The decay rate was defined as $d\,\Delta N/dt = -K \cdot \Delta N\,(t)$ and the lifetime was $1/\,K$. Our calculations allowed us to study dynamics of ΔN in the time window 0–1600 fs and to extrapolate the lifetimes. The simulated lifetimes (Supplementary Figure 6a) verified the reduction in lifetime with increased pump intensity and were qualitatively consistent with experiment (Figure 2). Moreover, even faster lifetimes were predicted for the dye molecules in close proximity to the Au NPs, which confirms the role of plasmons in enhancing the stimulated emission as well as absorption (Supplementary Figure 6b). Although experiments could not resolve temporally or spatially, calculations indicated that only dye molecules located close to Au NPs were efficiently involved in the energy transfer process and contributed to lasing action. The excess dye molecules introduced slow lifetime decay components that tended to obscure the fast components, which was consistent with the experimental observations.

Supplementary Note 2: Calculation of Gain and Loss

The quantum yield of IR-140 dye molecules in a uniform environment at room temperature is $\eta = \tau_{r0}^{-1}/(\tau_{r0}^{-1} + \tau_{nr}^{-1}) = 16\%$, where τ_{r0} and τ_{nr} are the intrinsic radiative and non-radiative decay lifetimes.[6] The radiative decay from a Purcell-enhanced spontaneous emission rate (F/τ_{r0}), where F is the Purcell enhancement factor can be estimated according to the following equations:[3]

$$\frac{1}{\tau_{uncoupled}} = \frac{1}{\tau_{r0}} + \frac{1}{\tau_{nr}}; \quad \frac{1}{\tau_{coupled}} = \frac{F}{\tau_{r0}} + \frac{1}{\tau_{r0}} + \frac{1}{\tau_{nr}} \quad \text{(Supplementary Equation 7)}$$

From the TCSPC lifetime measurement, we determined the slow time constant $\tau_{uncoupled}$ to be 715 ps and measured fast components of 21 ps (approaching threshold) and 12 ps (above threshold, limited by the resolution of TCSPC). Since stimulated emission induces fast decay, we used 21 ps as the fast time constant $\tau_{coupled}$ to avoid overestimation of the Purcell factor. We estimate that the Purcell factor F is ca. 200, in agreement with our previous work on Au NP arrays and solid gain media determining F from transient absorption (TA) measurements below threshold.[3] Note that TA is a pump-probe technique where the excited state population is monitored, and so dynamics of the energy transfer between dye molecules and the surface plasmons is indirectly determined. This F value (200) will be similar for other substrates and IR-140 in other solvents in this work. The cavity loss per length is given by $\omega n/cQ$, where n is the refractive index and Q is the quality factor.[7] For Au NPs arrays in different index environments, the loss was estimated to be 4580-4920 cm^{-1} using the quality factors (Q = 210-230) of the lattice plasmons measured in experiments. Using the emission cross section reported in the reference,[8] the gain was estimated to be $N\sigma_e$= 360 cm^{-1} (for 1 mM),[9] which cannot compensate the loss. With Au NPs arrays, however, the emission cross section $\sigma_e' = F\sigma_e$[10] can be enhanced by Purcell factor. With an F

estimated at 200, the cavity-enhanced gain is enough to overcome the loss and enable the lasing action.

Supplementary References

1. Zou, S. & Schatz, G. C. Narrow plasmonic/photonic extinction and scattering line shapes for one and two dimensional silver nanoparticle arrays. *J. Chem. Phys.* **121,** 12606–12612 (2004).
2. Henzie, J., Lee, M. H. & Odom, T. W. Multiscale patterning of plasmonic metamaterials. *Nature Nanotech* **2,** 549–554 (2007).
3. Zhou, W. *et al.* Lasing action in strongly coupled plasmonic nanocavity arrays. *Nature Nanotech* **8,** 506–511 (2013).
4. Dridi, M. & Schatz, G. C. Model for describing plasmon-enhanced lasers that combines rate equations with finite-difference time-domain. *J. Opt. Soc. Am. B* **30,** 2791–2797 (2013).
5. Nagra, A. S. & York, R. A. FDTD analysis of wave propagation in nonlinear absorbing and gain media. *IEEE Trans. Antennas Propagat.* **46,** 334–340 (1998).
6. Rurack, K. & Spieles, M. Fluorescence Quantum Yields of a Series of Red and Near-Infrared Dyes Emitting at 600-1000 nm. *Anal. Chem.* **83,** 1232–1242 (2011).
7. Amnon, Y. *Optical electronics in modern communications*. (Oxford University Press, 1997).
8. Sperber, P., Spangler, W., Meier, B. & Penzkofer, A. Experimental and theoretical investigation of tunable picosecond pulse generation in longitudinally pumped dye-laser generators and amplifiers. *Optical and Quantum Electronics* **20,** 395–431 (1988).
9. De Leon, I. & Berini, P. Amplification of long-range surface plasmons by a dipolar gain medium. *Nature Photonics* **4,** 382–387 (2010).
10. Suh, J. Y. *et al.* Plasmonic bowtie nanolaser arrays. *Nano Letters* **12,** 5769–5774 (2012).
